# Modulating Multisensory Processing: Interactions Between Semantic Congruence and Temporal Synchrony

**DOI:** 10.3390/vision9030074

**Published:** 2025-09-01

**Authors:** Susan Geffen, Taylor Beck, Christopher W. Robinson

**Affiliations:** 1 Department of Psychology, Occidental College, 1600 Campus Rd., Los Angeles, CA 90041, USA; sgeffen@oxy.edu (S.G.); taylorraybeck@gmail.com (T.B.); 2 Department of Psychology, The Ohio State University at Newark, 1179 University Dr., Newark, OH 43055, USA

**Keywords:** semantic congruency, cross-modal processing, modality dominance

## Abstract

Presenting information to multiple sensory modalities often facilitates or interferes with processing, yet the mechanisms remain unclear. Using a Stroop-like task, the two reported experiments examined how semantic congruency and incongruency in one sensory modality affect processing and responding in a different modality. Participants were presented with pictures and sounds simultaneously (Experiment 1) or asynchronously (Experiment 2) and had to respond whether the visual or auditory stimulus was an animal or vehicle, while ignoring the other modality. Semantic congruency and incongruency in the unattended modality both affected responses in the attended modality, with visual stimuli having larger effects on auditory processing than the reverse (Experiment 1). Effects of visual input on auditory processing decreased under longer SOAs, while effects of auditory input on visual processing increased over SOAs and were correlated with relative processing speed (Experiment 2). These results suggest that congruence and modality both impact multisensory processing.

## 1. Introduction

Most tasks involve the processing of auditory and visual information. For example, you may see and hear a dog when out for a morning walk. While much of the early research on perception and attention initially focused on only single sensory domains, within the last four decades, the focus has shifted to examining multisensory processing at the intersection of auditory and visual domains, e.g., [1,2,3,4,5,6,7,8,9,10,11]. Multisensory processing can often result in facilitation and/or interference effects [12,13], with some studies showing that visual stimuli affect auditory processing [2] and others showing that auditory stimuli affect visual processing [14]. While many studies have examined these issues, questions remain about the mechanisms underlying semantic congruency effects and modality dominance.

Semantic congruency (e.g., a car paired with a car horn) can facilitate processing, e.g., [13,15], whereas semantic incongruency (e.g., a dog paired with a car horn) can interfere with processing [16]. For example, congruent stimuli prompt faster (visual) recognition and response [17,18] and greater accuracy compared to incongruent stimuli [13]. Congruency is important. However, proper controls are needed in behavioral studies to determine which of several explanations, either individually or in combination, best explain the following results: (1) congruent multisensory stimuli are encoded more efficiently, (2) responses are faster on congruent trials because both auditory and visual modalities are associated with the same response (e.g., seeing a dog and hearing a cat both prompt an Animal response), and/or (3) responses to incongruent stimuli are slower due to disrupted encoding or post-perceptual factors (e.g., decision making).

Prior research suggests that semantic congruence can engage (spatial) attention even when audiovisual stimuli are task-irrelevant [19,20] and perceptual load is low [19]. These results suggest that semantic congruence does not automatically engage attention (bottom–up processing) but requires some top–down experience to guide attentional capture. Hirst et al. [21] used a variation on the Stroop task to examine the impact of semantic congruency on early (encoding) versus later stages of processing (response selection). Response congruent trials (different stimuli but same response) were slower than congruent trials, while incongruent trials had the slowest RT and decreased accuracy. Unimodal interference occurred at both the encoding and response selection stages, while cross-modal interference primarily occurred at the encoding stage. Post-perceptual processing (e.g., short-term memory retrieval) was facilitated by congruence between the target visual objects and the accompanying sound, and when the target object was consistent with the larger visual scene [22]. Long-term recognition memory is also facilitated by semantic congruence during the encoding phase for both younger and older adults [23]. Thus, as highlighted above, semantic congruency effects are complex and can occur at various points over the course of processing.

The current study used a variation of a semantic congruency task (discussed more below) to better understand the effects of multisensory presentation on auditory and visual processing. While semantic congruency tasks typically focus on facilitation effects (e.g., faster and more accurate responses on congruent trials), one novel component of the current study is that we also examined performance on incongruent trials to see which modality dominated when there was competition. More specifically, the current study required participants to quickly respond to auditory or visual information while ignoring competing information in the non-attended modality. Because both modalities were assessed (attend to auditory/ignore visual and attend to visual/ignore auditory), it is possible to determine whether competing auditory or visual information has a larger effect on processing. Thus, while the current study used a semantic congruency task to examine the facilitation effects, it also contributes to modality dominance research by determining which modality has a greater cost on processing in a second modality.

Research into modality dominance demonstrates that auditory and visual stimuli can have different effects, with auditory stimuli interfering with visual processing in some situations and visual stimuli interfering with auditory processing in others (see [7,10] for reviews). For example, when participants had to indicate if two picture–sound pairings were identical or different, the presence of an auditory stimulus slowed down visual responses and delayed the latency of first fixations compared to a unimodal visual baseline [14]. At the same time, the presence of the visual stimulus either had no effect or sped up auditory responding [24]. Interestingly, when the participants had to not only detect a change but also report what modality changed (picture or sound), modality dominance reversed to visual dominance [25]. The finding that visual dominance was only found when making separate responses to auditory and visual input is consistent with Colavita visual dominance effects where participants have to quickly respond to auditory and visual stimuli and often fail to report hearing a sound when it is paired with a visual stimulus [2,26], possibly suggesting that visual dominance effects occur later in the course of processing and are more susceptible to response demands [25].

Many mechanisms have been proposed to explain auditory and visual dominance (see [7,10] for reviews). One proposal for auditory dominance argues that auditory stimuli, due to their dynamic and transient properties, may automatically engage attention and are processed before shifting attention to the visual modality [7]. Support for this account comes from studies showing that the presence of an auditory stimulus slows down responses and fixations to visual stimuli, whereas adding a visual stimulus has no cost on auditory processing [24]. The law of prior entry [27] proposes a race model with visual dominance stemming from faster processing of visual information. If the direction of dominance stems from the modality that is faster at engaging attention, dominating initial processing, then it should be possible to reverse these effects by changing the relative timing of auditory and visual information. While some studies have shown that modality dominance can be reversed by manipulating stimulus order [28], others have shown that you can lessen visual dominance by delaying the onset of visual stimuli but not eliminate it [29]. Finally, it is also possible that visual dominance stems from sensory modalities being inhibitory in nature, with vision being more likely to inhibit other modalities, since approximately 50% of the brain is dedicated to the visual system [10,30,31]. According to this account, the visual modality should win in most contexts.

**Figure 1:**
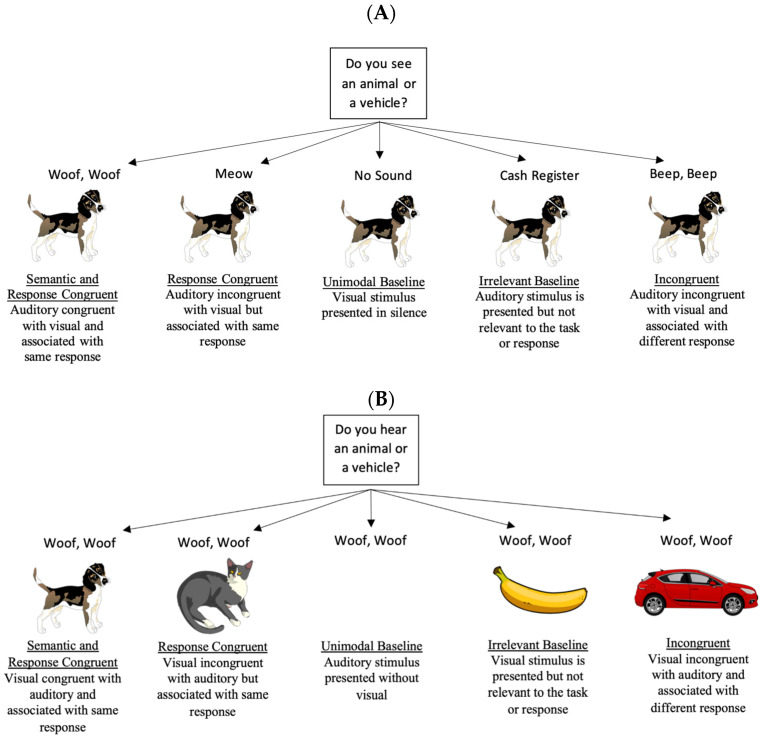
Examples of trial types across the Attend to Visual (**A**) and Attend to Auditory (**B**) conditions. The correct response across all trial types reported in Figure 1 is to press the animal button.

The current research used a Stroop-like task [32] to better understand the mechanisms underlying modality dominance and semantic congruency effects. Participants in Experiment 1 were presented with audiovisual information that was either congruent, incongruent, or irrelevant, and participants had to quickly determine if the information in the attended modality (e.g., auditory) was an animal or a vehicle (see Figure 1 for trial types). Expanding on previous research, which normally studies congruency within a sensory modality or how congruency in one modality affects processing in another modality (though see [21]), the current research examined how congruent and incongruent auditory stimuli affect visual processing and how congruent and incongruent visual stimuli affect auditory processing. Thus, these experiments examined which modality (auditory or visual) has a stronger effect on multisensory processing.

We predict that participants should demonstrate faster response times and greater accuracy when responding to congruent versus incongruent stimuli. Unimodal control trials (just picture or sound) will help determine if these effects stem from congruent stimuli being encoded faster or from incongruent stimuli slowing down processing/responding. In regard to modality dominance, auditory stimuli should have a larger effect on visual processing if auditory stimuli automatically engage attention and delay visual processing [7], whereas stronger visual effects would be consistent with visual dominance research in adults, with approximately 50% of the brain dedicated to vision [10,30,31]. Finally, Experiment 2 manipulated the relative timing of auditory and visual information. If modality dominance effects stem from the modality that is faster to engage attention (i.e., winning the race) dominating the other modality [27], then presenting the non-dominant modality first should reverse the effects.

## 2. Experiment 1

### 2.1. Materials and Methods

#### 2.1.1. Participants

Forty-five participants completed the study. The sample size is consistent with previous behavioral research examining cross-modal processing in adults [21,29]. The data exclusion criteria included if participants missed at least half of the attention checks during the auditory block (*n* = 4) or experimenter error (*n* = 5), leaving 36 participants in the final sample. All participants were recruited from Occidental College or The Ohio State University at Newark, all self-reported normal hearing and normal or corrected to normal vision, English-speaking, and all were over 18. Participants received course credit and provided consent before the start of the experiment. Experimental procedures were approved by the Occidental College Human Subjects Institutional Review Board (Protocol Number FA21-28-GEF) or The Ohio State University IRB (2021B0350).

Participants in the final sample had a mean age of 19.28 years (*SD* = 1.26, *range* 18–23 years). Twenty participants were female (including transgender women), 14 were male (including transgender men), and two chose not to answer. Nineteen participants were White, six were Black or African American, five were Asian, four were Other/Multiracial, and two chose not to answer.

#### 2.1.2. Stimuli and Design

The visual stimuli for the experiment consisted of various images of familiar animals (e.g., frog, elephant), vehicles (e.g., boat, motorcycle), and household objects (e.g., clock, apple; see Figure 1 for examples), which were taken from https://pixabay.com, as well as a green circle which was created in Photoshop with a transparent background and served as a visual attention check. The images were presented centrally on a computer monitor at approximately 600 × 600 resolution. Auditory stimuli consisted of 500 ms clips of the corresponding sounds taken from the Marcell sound library [33] and were edited in Audacity. The sounds were presented via headphones at approximately 65 dB. The stimuli had to meet several characteristics: (a) the visuals had to have a similar cartoon-like style, (b) auditory/visual stimuli had to be easily recognizable (even under 500 ms stimulus presentations), and (c) come from highly familiar categories. See Appendix A for a full list of stimuli.

Modality (**Attend To Visual** vs. **Attend To Auditory**) and stimulus/response congruency were manipulated within subjects. **Attend To Visual** and **Attend To Auditory** trials were blocked, and the block order was counterbalanced across participants. In the **Attend To Visual** block, participants had to ignore what they heard and quickly determine if each visual stimulus was an animal or a vehicle. In the **Attend To Auditory** block, participants had to ignore the visual stimulus and determine if they heard an animal or vehicle sound. The trial order within each block was randomized for each participant. The five trial types were *Semantic and Response Congruent*, *Response Congruent*, *Unimodal*, *Irrelevant, and Incongruent*. There were 48 trials for each of the five trial types, with 240 **Attend To Visual** trials and 240 **Attend To Auditory** trials, for a total of 480 trials (see Figure 1 for trial types and examples). Auditory and visual stimuli were presented for 500 ms and shared the same onset and offset. Then, the screen remained blank until the participants made a response, after which the next trial would begin. There were also six attention checks per block (12 total), consisting of a green circle that appeared in the center of the screen with no accompanying audio. This ensured that the participants stayed focused on the screen throughout the entire experiment, especially during the **Attend To Auditory** trials, where participants could just shut their eyes to avoid the conflicting visual information.

Participants indicated an animal stimulus by pressing one button (either the “*p*” or “q” key) and indicated a vehicle stimulus by pressing the other button. The correspondence between keys and category (animal or vehicle) was counterbalanced across participants. Attention checks (green circle) required a space bar press.

The experiment was conducted using the software package PsychoPy (v2021.2.3) [34]. For the participants at Occidental College, the program was installed and run on the research assistants’ computers. For participants at The Ohio State University, the study was completed on one of the computers in the Multisensory Processing Across Development (MAD) lab. Participants at both schools were provided with over-ear headphones to complete the study. The reaction time and accuracy were recorded for each trial.

#### 2.1.3. Procedure

All participants were recruited at either Occidental College or The Ohio State University at Newark. Upon arrival, participants entered a quiet room with a computer and minimal distractions, where they provided consent for the study. Next, participants answered a series of demographic questions (e.g., age, gender) and read the specific instructions about the procedure of the experiment prior to the first trial of each block. For example, participants would see the following instructions at the start of the **Attend to Auditory** block: “Remember, you will hear sounds and see pictures at the same time. In this part of the study, you have to report what you hear”. They were also informed which button corresponded to each category. Half the participants pressed “*p*” for animals and “q” for vehicles; the buttons were counterbalanced for the other half of the participants. Each block began with four practice trials to ensure that the participants understood the instructions and procedure. Participants could take a break halfway through the experiment between the two blocks. The whole procedure lasted approximately 30 min.

### 2.2. Results

**Figure 2:**
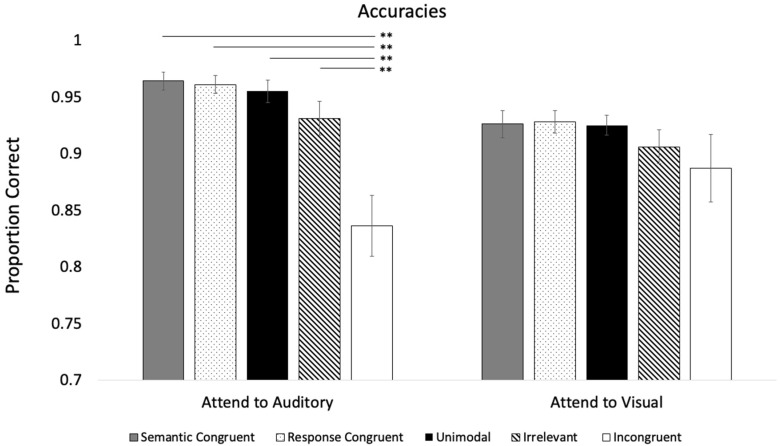
Proportion of correct responses across trial types and conditions in Experiment 1. Solid bars are associated with Semantic Congruent, Unimodal, and Incongruent trials. Patterned bars are additional controls. Error bars denote standard errors, and “*” and “**” denote that the two means differ, *p* < 0.05 and 0.001, respectively.

Participants detected 96% of the attention check trials in the **Attend to Auditory** condition and 88% in the **Attend to Visual** condition. Thus, the participants in both conditions attended to the target and non-target modalities. Accuracies across Modality and Trial Types are presented in Figure 2. With the exception of the *Response Congruent* mean in the **Attend to Auditory** Condition, the other means significantly deviated from a normal distribution, Shapiro–Wilk test, *p* < 0.003. Thus, we reported the median response times for both experiments.

The Modality × Trial type repeated-measures ANOVA revealed a main effect of Trial Type, *F* (4, 140) = 16.24, *p* < 0.001, η_p_^2^ = 0.32, and post hocs, with Bonferroni adjustments revealing that accuracy on the *Incongruent* trials (*M* = 0.86, *SE* = 0.02) was significantly lower than all of the other trial types (*M*’s > 0.92), *p* < 0.001, η_p_^2^ > 0.32. The analysis also revealed a Modality × Trial Type interaction, *F* (4, 140) = 3.90, *p* = 0.005, η_p_^2^ = 0.10. As shown in Figure 2, simple main effects revealed that the different auditory trial types did not affect visual processing, *p* = 0.206 (see right side of Figure 2). In contrast, the different visual trials affected auditory processing, *p* < 0.001 (see left side of Figure 2), with *Incongruent* trials differing from the other trial types. The main effect of Modality was not significant, *F* (1, 35) = 1.40, *p* = 0.245, η_p_^2^ = 0.04.

**Figure 3:**
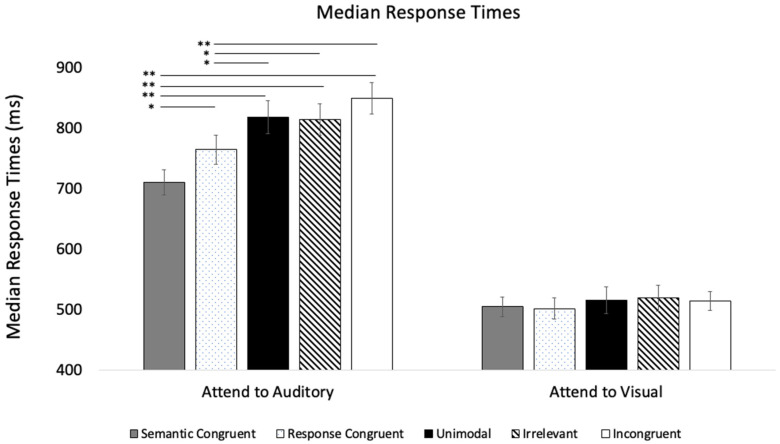
Median response times across trial types and conditions in Experiment 1. Solid bars are associated with Semantic Congruent, Unimodal, and Incongruent trials. Patterned bars are controls. Error bars denote standard errors, and “*” and “**” denote that the two means differ, *p* < 0.05 and 0.001, respectively.

The median RT analyses point to the same processing asymmetry, with visual stimuli having a larger effect on auditory processing than vice versa (see Figure 3 for means). The Modality × Trial Type repeated-measures ANOVA revealed a main effect of Modality, *F* (1, 35) = 198.56, *p* < 0.001, η_p_^2^ = 0.85. As shown in Figure 3, the visual RTs (*M* = 510.67, *SE* = 18.06) were faster than the auditory RTs (*M* = 790.88, *SE* = 22.90). The analysis revealed a main effect of Trial Type, *F* (4, 140) = 22.46, *p* < 0.001, η_p_^2^ = 0.39. Post hoc tests with Bonferroni adjustments revealed that *Semantic and Response Congruent* trials marginally differed from *Response Congruent* trials, *p* = 0.051, and from all of the other trial types, *p* < 0.001, and the *Response Congruent* trials also differed from the *Unimodal*, *Irrelevant*, and *Incongruent* trial types, *p* < 0.011. There was also a Modality × Trial Type interaction, *F* (4, 140) = 17.65, *p* < 0.001, η_p_^2^ = 0.34, and simple effects revealed that visual trial types affected auditory response times, *p* < 0.001, whereas auditory trial types had no effect on visual response times, *p* = 0.119.

### 2.3. Discussion

Experiment 1 reveals several important findings regarding the possible mechanisms underlying the semantic congruency effects and the relative weights of auditory and visual information on these effects. The accuracy and response time data both show an important asymmetry, with visual stimuli having a larger effect on auditory processing than the reverse, consistent with visual dominance, e.g., [10,21]. This suggests that visual input contributes more to semantic congruency than auditory information, which may stem from several factors. First, visual response times were significantly faster than auditory response times. Thus, it is possible that participants had already made their decision about the visual stimulus before processing the sounds [27]. Second, auditory and visual systems may be inhibitory in nature. Given that approximately 50% of the brain is dedicated to vision, activating the visual system may attenuate auditory processing [10] and decrease the effects of auditory stimuli on semantic congruency (e.g., the sounds were not fully processed, thus weakening facilitation and interference effects).

The findings also provide insight into the mechanisms underlying semantic congruency and incongruency. While visually incongruent stimuli decreased auditory accuracy compared to the *Unimodal* trials, visually congruent stimuli sped up the auditory responses. Regarding interference effects, this suggests that the presence of a conflicting visual stimulus (e.g., hearing a meow but seeing a train) causes interference. Additional support for this claim is that the irrelevant visual stimulus (e.g., hearing meow but seeing a banana) had no effect, suggesting that it was the conflict that made the decision-making process more challenging. If these effects resulted from attentional factors with visual stimuli, in general, pulling attention away from the auditory modality [35] or from visual stimuli inhibiting auditory processing, then the visual stimuli in the irrelevant condition should have also hindered performance.

One proposal underlying semantic congruency is that these congruent multisensory stimuli are processed more efficiently than the same stimulus presented to a single modality, possibly due to the automatic binding across sensory modalities and/or increased saliency of the congruent information [36]. It is important to note that semantic congruency and response congruency both had effects on auditory processing, with response congruency speeding up responses relative to the unimodal condition and semantic congruency having additional effects. This finding may indicate that semantic congruency speeds up encoding, while response congruency may also speed up later stages of processing (e.g., the decision or the response).

## 3. Experiment 2

Experiment 2 replicates and extends Experiment 1 by evaluating the impact of visual and auditory asynchrony on semantic congruency and whether these effects are symmetrical across sensory modalities. Recall that unimodal visual processing was faster than unimodal auditory processing in Experiment 1. If stronger effects of visual input on auditory processing stem from visual stimuli being faster to engage attention, these effects should reverse when auditory stimuli are presented before visual stimuli. Alternatively, presenting the auditory stimulus before the visual stimulus may have little effect if modality dominance stems from sensory inhibition [10], more efficient visual selective attention, and/or better filtering of auditory distractors.

### 3.1. Materials and Methods

#### 3.1.1. Participants

Forty participants completed the study. The sample size is consistent with previous behavioral research examining cross-modal processing in adults [21,29], and it is also consistent with research manipulating the stimulus onset asynchrony of auditory and visual information [37]. The data exclusion criteria included if the participants missed at least half of the attention checks during the auditory block (*n* = 3) or had an incomplete data set (*n* = 1), leaving 36 participants in the final sample. All participants were recruited from Occidental College or The Ohio State University at Newark, self-reported normal hearing and normal or corrected to normal vision, English-speaking, and were over the age of 18. Participants received course credit and provided consent online before the experiment began.

Participants in the final sample had a mean age of 19.88 years (*SD* = 1.75, *range =* 18–25). Twenty-nine were female (including transgender women), six were male (including transgender men), and one chose to self-describe. Fifteen participants were White, nine were Black or African American, seven were Asian, and five were Other/Multiracial.

#### 3.1.2. Stimuli, Design, and Procedure

Experiment 2 was identical to Experiment 1 with the following exceptions. We only included *Semantic and Response Congruent (Congruent)*, *Unimodal,* and *Incongruent* trials to keep the task to around 30 min in duration. Auditory and visual stimuli kept the same duration (500 ms), but no longer shared onset and offset times. To try to reverse visual dominance, for both blocks, we randomly presented the auditory stimulus either 0, 150, 300, or 450 ms before the visual stimulus. We used 150 ms onset intervals, which mirror those used by [28], and, given the previous literature indicating a critical period around 300 ms where larger intervals make audiovisual integration difficult [38,39], it was logical to extend the onset range to 450 ms.

**Figure 4:**
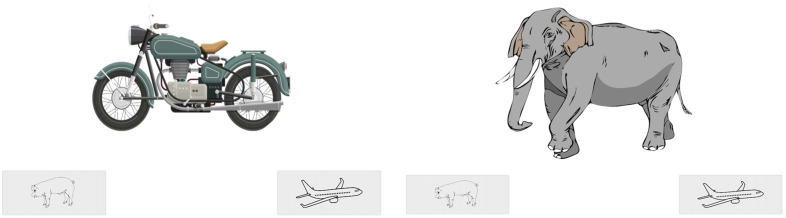
Examples of practice trial types with touchscreen buttons in the Attend to Visual condition. The correct response is to press the vehicle button (**left**) and to press the animal button (**right**). A circle was presented between the two buttons for attention check responses in the Attend to Auditory condition, and a phone was presented between the two buttons for attention check responses in the Attend to Visual condition.

We also made several changes to the experiment to gradually move over to an online study. First, the experiment was administered on the website Pavlovia.org. Second, the participants recruited from The Ohio State University completed the experiment in a laboratory on a 22″ Planar PXL2230 1920 × 1080 touchscreen monitor. The participants recruited from Occidental College completed the experiment on a personal computer from home. Third, participants responded by pressing (touchscreen) or clicking (mousepad) an animal button (a pig) or a vehicle button (a plane) on the screen (see Figure 4). Button location (bottom left vs. bottom right) was randomized for each participant and counterbalanced across participants. Fourth, the instructions before each block were presented by video rather than printed on the screen.

Finally, in Experiment 1, we included visual attention checks during both the **Attend to Auditory** and **Attend to Visual** blocks. This meant that participants had to allot even some attention to both modalities in the **Attend to Auditory** block, but all trials (test and attention checks) were in the visual modality for the **Attend to Visual** block. To address this potential bias, in Experiment 2, we used visual attention check trials (green circle) during the **Attend To Auditory** block and auditory attention checks (phone sound) during the **Attend To Visual** block. Participants had to respond by pressing the appropriate button (either a green circle or phone). There were 96 of each trial type per block, plus six attention checks, totaling 294 trials per block and 588 trials overall. There were also eight practice trials before each block to introduce participants to all the SOAs.

### 3.2. Results

**Figure 5:**
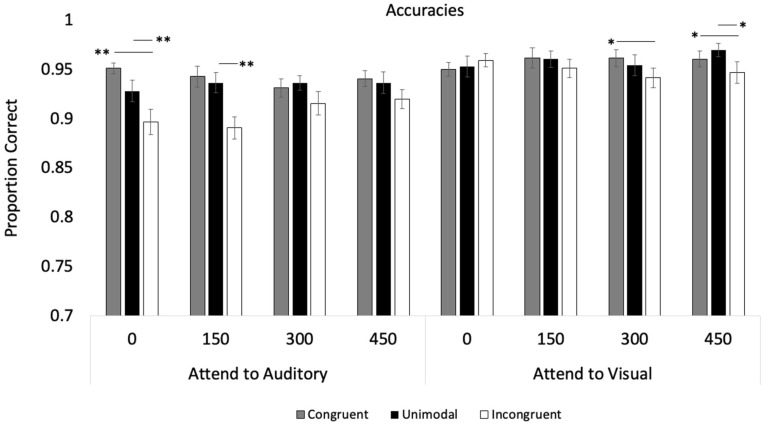
Proportion of correct responses across trial types, SOA, and conditions in Experiment 2. Error bars denote standard errors, and “*” and “**” denote that the two means differ, *p* < 0.05 and 0.001, respectively.

Participants detected 96% of the attention check trials in the **Attend to Auditory** condition and 97% in the **Attend to Visual** condition. Thus, as in Experiment 1, the participants in both conditions attended to the target and non-target modalities. Accuracies were submitted to a Modality (2) × Trial Type (3) × SOA (4) repeated-measures ANOVA (see Figure 5 for means and Table 1 for statistics). The analysis revealed a main effect of Trial Type, *p* < 0.001, and post hocs with Bonferroni adjustments revealed that accuracy on *Incongruent* trials (*M* = 0.95, *SE* = 0.01) was significantly lower than the other trial types (*M*’s > 0.97), *p* < 0.001. The analysis also revealed a Modality × Trial Type interaction, *p* = 0.044, and the Modality × Trial Type × SOA interaction was also significant, *p* < 0.001.

The three-way interaction was broken down by conducting two separate Trial Type × SOA repeated-measures ANOVAs, one for each modality. When examining the effects of auditory distractors on the visual response accuracies, the analysis only revealed an effect of Trial Type, *p* = 0.036, with the *Incongruent* condition (*M* = 0.96, *SE* = 0.01) differing from the *Congruent* condition (*M* = 0.97, *SE* = 0.01), *p* = 0.041. See Table 1 for statistics. There was no evidence that the *Congruent* or *Incongruent* conditions differed from the *Unimodal* condition, *p* > 0.170. In contrast, the analysis examining the auditory responses revealed a main effect of Trial Type, *p* < 0.001, and a significant Trial Type × SOA interaction, *p* = 0.011. As can be seen in Figure 5, while the trial type had an effect at SOAs of 0 and 150, *p* < 0.001, this effect disappeared at SOA 300, *p* = 0.147, and 450, *p* = 0.631.

**Figure 6:**
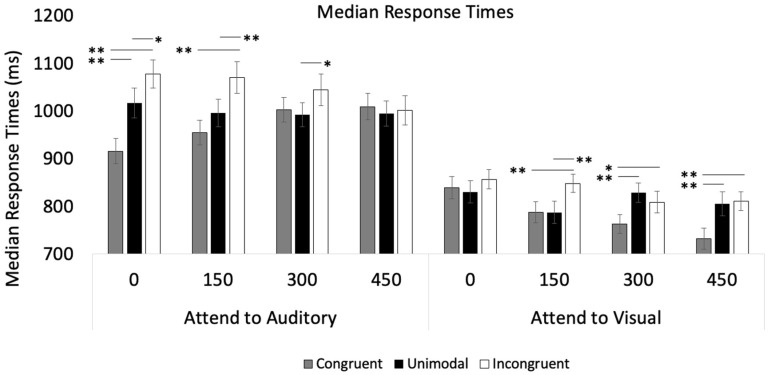
Median response times across trial types, SOA, and conditions in Experiment 2. Error bars denote standard errors, and “*” and “**” denote that the two means differ, *p* < 0.05 and 0.001, respectively.

Median RTs were also submitted to a Modality × Trial Type × SOA repeated-measures ANOVA, which revealed a main effect of Trial Type, *p* < 0.001 (see Figure 6 for means and Table 2 for statistics). Post hoc tests with Bonferroni adjustments revealed that *Congruent* trials (*M* = 875.06, *SE* = 20.86) were faster than *Unimodal* trials (*M* = 912.87, *SE* = 22.27), and *Unimodal* trials were faster than *Incongruent* trials (*M* = 939.46, *SE* = 23.04), *p* < 0.001. The analysis also revealed a main effect of Modality, *p* < 0.001. As shown in Figure 6, visual RTs (*M* = 812.35, *SE* = 19.75) were faster than auditory RTs (*M* = 1005.25, *SE* = 25.78). The main effect of SOA was also significant, *p* < 0.001. Post hoc tests with Bonferroni adjustments show that the response times in the SOA 450 condition (*M* = 891.88, *SE* = 21.35) significantly differed from both the SOA 0 condition (*M* = 922.44, *SE* = 23.12), *p* < 0.001, and the SOA 150 condition (*M* = 916.06, *SE* = 22.76), *p* = 0.006.

Except for the Modality × Trial Type interaction (*p* = 0.059), all the two-way and three-way interactions were significant (see Figure 6 and Table 2). As in the accuracy analyses, the three-way interaction was broken down by conducting two separate Trial Type × SOA repeated-measures ANOVAs, one for each modality. The analysis examining visual RTs revealed that both the main effects and the SOA × Trial Type interaction were significant, *p* < 0.001. As indicated in Figure 6, the effects of auditory trial types increased as SOA increased, with no effects of auditory input on visual responses at SOA 0, *p* = 0.233, while having significant effects at SOAs of 150, 300, and 450, *p* < 001. The analysis examining the effects of the visual input on auditory RTs shows a main effect of Trial Type and a significant Trial Type × SOA interaction, *p* < 0.001 (see Table 2 for statistics). In contrast to the **Attend to Visual** condition, the effects of the visual input on auditory RTs were stronger with shorter SOAs and decreased under longer SOAs (see Figure 6). More specifically, the effects of Trial Type were significant at SOAs of 0 and 150, *p* < 0.001, attenuated at SOA 300, *p* = 0.015, and disappeared at SOA 450, *p* = 0.750.

#### Exploratory Analyses

Additional analyses examined a potential factor that might predict which individuals will show the strongest semantic congruency and incongruency effects. Note that the effects of visual input on auditory processing/responding were strongest under shorter SOAs. Thus, we combined data from Experiment 1 with the SOA 0 condition in Experiment 2 to determine if relative processing speed (unimodal visual RT minus unimodal auditory RT) could predict the effects of visual semantic congruency and incongruency on auditory processing. If cross-modal effects stem from the modality that is processed faster, dominating the other modality, then the effects of visual input on auditory processing should be stronger in participants who are faster at processing visual compared to auditory stimuli (negative scores). See [7,27] for related discussions, whereas the effect of auditory input on visual processing should be stronger in participants who are faster at processing auditory information relative to visual.

**Figure 7:**
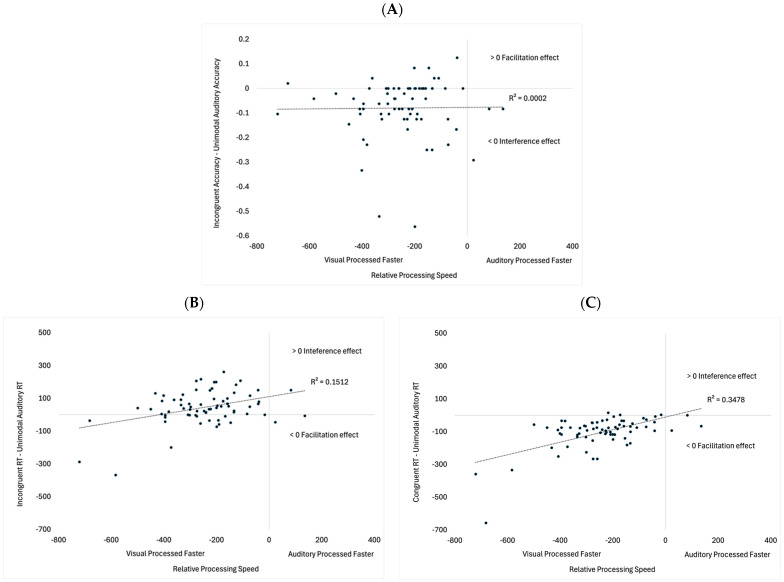
Scatterplots examining the relationships between relative processing speed and costs on Incongruent accuracy (**A**), Incongruent RT (**B**), and facilitation on Congruent RT (**C**). The diagonal lines represent trend lines. Note that the patterns did not change when removing outliers +/− 3 *SD*: (**A**) *r* (68) = −0.01, *p* = 0.989, (**B**) *r* (69) = 0.310, *p* = 0.009, (**C**) *r* (69) = 0.52, *p* < 0.001.

The following analyses examined whether relative processing speed (unimodal visual RT minus unimodal auditory RT) could predict which participants would show (a) the most significant drop in accuracy on the auditory incongruent trials (incongruent accuracy minus unimodal auditory accuracy), (b) the greatest slowdown in response times on the auditory incongruent trials (incongruent RT minus unimodal auditory RT), and (c) the strongest facilitation effect on the auditory congruent trials (congruent RT minus unimodal auditory RT). See Figure 7 for scatterplots. As can be seen in Figure 7A, there was no correlation between the relative processing speed and the accuracy costs on the *Incongruent* trials, *r* (70) = −0.01, *p* = 0.913. As shown in Figure 7B, relative processing speed correlated with slower response times on the *Incongruent* trials, *r* (70) = 0.39, *p* < 0.001, with incongruent visual stimuli having greater costs for participants with comparable processing speeds. Note that only three of the 72 participants were faster at processing the sounds. Thus, it is unclear if this trend would have continued in faster auditory processors. Finally, relative processing speed also correlated with facilitation effects on *Congruent* trials, *r* (70) = 0.59, *p* < 0.001 (see Figure 7C). While most participants showed faster responses on the *Congruent* trials, these effects were stronger in participants who showed faster visual processing speeds relative to auditory.

The primary goal of Experiment 2 was to replicate and extend the results from Experiment 1 by evaluating the impact of visual and auditory asynchrony on semantic congruency. Mirroring Experiment 1, the response times and accuracy rates in Experiment 2 demonstrated an asymmetry with the visual stimuli having a larger effect on auditory processing than the reverse (see Figure 5). However, the effects of visual input on auditory accuracy only occurred when SOAs were short (0, 150 ms). Regarding response times, congruent visual stimuli sped up auditory responding, and incongruent visual stimuli slowed auditory responding. However, these effects also attenuated as the SOA increased (see Figure 6). Finally, while the effects of visual stimuli on auditory processing decreased over the SOAs, the effects of auditory input on visual processing increased over longer SOAs.

Exploratory analyses examined whether relative processing speed could predict the effects of visual input on auditory congruency and incongruency effects. Relative processing speed was not correlated with decreased accuracy on the *Incongruent* trials. However, it did correlate with response times on *Congruent* and *Incongruent* trials. Regarding incongruency effects, visual incongruency had a larger effect on participants who had more similar processing speeds across sensory modalities (see Figure 7B). Finally, visual congruency had stronger effects in participants who were faster at processing the visual information, relative to the auditory information (see Figure 7C).

## 4. General Discussion

The current experiments examined how congruency affects multisensory processing and responding. Unlike many previous studies, the current research examined both the effects of auditory congruency on visual processing and visual congruency on auditory processing, thus contributing to the research on modality dominance. Experiment 1 incorporated additional control trials to shed light on mechanisms underlying modality dominance and semantic congruency. Finally, stimulus onset was manipulated in Experiment 2 to determine if the direction of modality dominance could be reversed by presenting the losing modality before the dominant modality.

Under simultaneous presentation, visually congruent and incongruent stimuli had a larger effect on auditory processing than vice versa. Both experiments found that incongruent stimuli interfered with participants’ multisensory processing, decreasing the accuracy on these trials compared to the *Unimodal* trials, with the greatest cost to those individuals with comparable visual and auditory processing speeds (correlational analyses in Experiment 2). By contrast, semantic congruency sped up responding, with faster responses on the *Semantic and Response Congruent* trials (Experiment 1) and *Congruent* trials (Experiment 2) compared to the *Unimodal* trials. Correlational analyses show that *Congruent* trials had the greatest facilitation effects for those with faster visual processing speeds relative to auditory.

The current research did not use a traditional visual dominance task and focused on auditory- and visual-based errors made on multimodal trials [2]. However, it did assess how auditory congruency affects visual processing and how visual congruency affects auditory processing. Under simultaneous presentation and short SOAs, visual stimuli affected auditory processing more than the reverse. In other words, visual input contributed to congruency effects more than sounds, which is consistent with the idea of visual dominance [10]. This finding can be explained by numerous accounts underlying visual dominance. However, as discussed in more detail below, the correlational and SOA analyses show that stimulus timing matters, which appears to provide support for prior entry [27]. The findings also conflict with the notion that sounds automatically grab attention and slow down or disrupt visual processing, a potential mechanism underlying auditory dominance [7]. We revisit this issue in the Section Limitations and Future Directions.

The current study had numerous controls in Experiment 1 to better understand modality dominance and congruency effects. For example, in the *Irrelevant* control trials, an animal or vehicle was paired with an irrelevant sound or picture, which was not associated with either response (e.g., the sound of a violin). If any auditory stimulus automatically grabs attention and pulls attention away from the visual modality [7], then interference should have also been found on the *Irrelevant* trials, which was not the case. Using the same logic, it is important to note that irrelevant visual stimuli (e.g., a banana paired with a dog bark) did not affect auditory processing, suggesting that decreased accuracy on the *Incongruent* trials stemmed from stimulus competition, not the need to process an additional stimulus.

By using *Semantic and Response Congruent* trials and *Response Congruent* trials, the current study also provides insight into the potential mechanisms underlying congruency effects. Semantic congruency can often lead to faster and more accurate processing [13]. However, this can stem from auditory and visual congruent stimuli being *encoded* more efficiently or from auditory or visual stimuli both being associated with the same *response*—see also [21] for a related discussion. One important finding is that *Response Congruent* trials sped up responding compared to the *Unimodal* condition, and semantic congruency had additional effects over and above response congruency. This suggests that post-perceptual processing may also underlie the facilitation effects and that caution is needed when speculating about the mechanisms underlying the semantic congruency effects when there is no response congruency control.

The SOA and correlational analyses both show that timing is important. At a general level, the effects of visual input on auditory processing were strongest under simultaneous presentation and weakened when the auditory stimulus was presented before the visual stimulus. At the same time, the decreased visual effects over longer SOAs were replaced by stronger auditory effects. The correlation analyses also examined the effects of stimulus timing by quantifying how quickly each participant responded to the unimodal auditory and visual information. Only three of the 72 participants processed the sounds faster than the images, which may explain why visual dominance was found. It is also important to note that performance on the task was associated with relative processing speed. Interference effects were stronger when auditory and visual processing speeds were more comparable (Figure 7B), and facilitation effects were stronger when participants were faster at processing the visual input relative to the sounds (Figure 7C). Visual dominance may stem from participants allocating more attentional resources to the visual modality because visual stimuli are less likely to engage attention compared to sounds [40]. However, in agreement with Posner et al. [40], an attentional bias may work in conjunction with prior entry [27], which would explain why the current study found that these effects change across SOA and are related to individual differences in processing speed.

The current findings also need to be reconciled with previous research manipulating SOA. In the current study, a reversal starts with longer SOAs (300, 450), where the impact of visual stimuli on auditory processing decreases, while the impact of auditory stimuli on visual processing starts to increase. Ciraolo et al. [37] showed the opposite pattern of modality dominance, with participants demonstrating auditory dominance when auditory stimuli preceded visual stimuli and when multimodal stimuli were presented simultaneously, and only showed visual dominance when the visual stimuli preceded the auditory by 200 ms. This difference in default modality dominance may be attributable to task differences, with Ciraolo et al. [37] using a perceptual task with novel stimuli while we used a categorical task with familiar stimuli and categories. Despite the differences in initial modality dominance, both studies suggest that timing matters, as the non-dominant modality had to precede the dominant modality by 200–300 ms before the dominance started to shift. Previous studies suggested this as a critical point for a breakdown of multisensory integration, though integration was typically more stable when visual stimuli preceded auditory stimuli [38,39].

### Limitations and Future Directions

While the current study included multiple controls to distinguish between the different mechanisms underlying semantic congruency and modality dominance, one limitation of the current research is that participants completed the study on a computer rather than on an eye tracker. For example, the current study found that response times were faster on the *Semantic and Response Congruent* trials compared to the *Response Congruent* trials, which may suggest more efficient encoding. However, it is also possible that this facilitation effect stemmed from attentional factors. Combining behavioral and eye tracking data may help differentiate the attentional, processing, and decision-making mechanisms underlying multisensory integration and modality dominance. For example, if intersensory interactions affect attention and speed up or slow down stimulus detection, then the latency of first fixation to a stimulus should occur earlier or later in the congruent and incongruent conditions, respectively [41]. Congruence and incongruence can also speed up or slow down processing time, which may be independent of stimulus detection and post-perceptual stages of processing. In this scenario, changes in response times should correlate with mean fixation durations. Finally, it is possible that incongruent stimuli have no effect on stimulus detection or processing speed but increase response times by affecting the decision stage of processing. If this is the case, then facilitation and interference effects should not correlate with the duration or latency of fixations, and increased cognitive load on incongruent trials should correlate with increased pupil size [42].

Another limitation relates to using strictly the visual attention checks in Experiment 1, which could contribute to visual dominance. However, as mentioned above, Experiment 2 balanced the modality of attention check trials, ensuring participants had to allot attention to both modalities in all trials (with the exception of the *Unimodal* trials). Experiment 2 in the current manuscript and a follow-up eye tracking study using the *Congruent, Incongruent,* and *Unimodal* stimuli from Experiment 1 found consistent results, with visual stimuli having a greater impact on auditory processing than the reverse. Thus, it is unlikely that the visual attention checks alone contributed to faster responses on visual compared to auditory trials.

The current study also used highly familiar stimuli and categories, with stimulus familiarity potentially affecting processing speed and modality dominance effects. Thomas et al. [13] suggested that familiar auditory stimuli speed up the processing of visual stimuli (see also [43] for a similar finding). At the same time, stimulus familiarity effects are complex and interact with task and age. For example, while familiar stimuli are more likely to dominate less familiar stimuli in 4-year-olds [44], unfamiliar stimuli in infants are associated with stronger auditory dominance [43]. One potential way to resolve this discrepancy is to posit that auditory stimuli disrupt the encoding of visual perceptual details but may not affect recognition of familiar visual categories (see also [45] for a similar discussion). However, this will need to be tested in future research.

Expanding on the idea that auditory stimuli may disrupt encoding but not the recognition of familiar categories, it will also be important to examine semantic congruency effects using novel stimuli/categories. First, it is well established that processing of the gist or category information occurs before processing the details of a stimulus [46]. For example, we classify an object as a dog before we process the individual markings and unique features of that dog, and once categories are learned, we can optimize our attention and focus on the features that define the category [47]. While labeling objects can direct attention to these category-relevant features [48], it is unclear how attentional weights to auditory and visual information change during learning. Using novel stimuli and novel categories will be important for capturing the contributions of auditory and visual information underlying semantic congruency *while* the categories are being formed, but may also be useful for examining developmental shifts since the stimuli and categories will be equally familiar across age, which is not the case when using familiar stimuli. Future research will need to examine whether novel stimuli (e.g., picture or sound of an alien creature) impose greater cognitive demands than familiar stimuli (e.g., picture of a dog), leading to increased processing costs, diminished performance, and possibly, a reversal in modality dominance.

## 5. Conclusions

In summary, semantic congruence and modality both impact multisensory processing. Visual stimuli have a greater impact on auditory processing when stimuli are presented synchronously, consistent with visual dominance [10]. Across both experiments, congruent stimuli lead to greater accuracy and faster RT, while incongruent stimuli lead to lower accuracy and slower RT. Our results align with prior research showing facilitation with semantic congruence and interference with incongruence [13,16]. We also expanded on prior research by examining the additional impact of timing. The reversed patterns in RT from short to long SOAs support the law of prior entry, where presenting the non-dominant modality before the prior can diminish or reverse modality dominance [27]. Congruency and incongruency effects were also correlated with individual differences in the relative processing speed of auditory and visual information, further highlighting the dynamics of multisensory processing.

## Figures and Tables

**Table 1:** Statistics from the Modality × Trial Type × SOA repeated-measures ANOVA when analyzing accuracy data.

Primary Accuracy Analyses	ANOVA (df) *F*	*p*	η_p_^2^
Modality	*F* (1, 35) = 2.09	=0.160	0.06
Trial Type	*F* (2, 70) = 26.95	<0.001	0.44
SOA	*F* (3, 105) = 0.86	=0.466	0.02
Modality × SOA	*F* (3, 105) = 0.72	=0.541	0.02
Modality × Trial Type	*F* (2, 70) = 3.26	=0.044	0.09
SOA × Trial Type	*F* (6, 210) = 0.58	=0.749	0.02
Modality × SOA × Trial Type	*F* (6, 210) = 4.18	<0.001	0.11
Attend to Visual			
Trial Type	*F* (2, 70) = 3.49	=0.036	0.09
SOA	*F* (3, 105) = 0.49	=0.691	0.01
SOA × Trial Type	*F* (6, 210) = 1.31	=0.254	0.04
Attend to Auditory			
Trial Type	*F* (2, 70) = 18.33	<0.001	0.34
SOA	*F* (3, 105) = 1.05	=0.375	0.03
SOA × Trial Type	*F* (6, 210) = 2.84	=0.011	0.08

**Table 2:** Statistics from the Modality × Trial Type × SOA repeated-measures ANOVA when analyzing response time data.

Primary Response Time Analyses	ANOVA (df) *F*	*p*	η_p_^2^
Modality	*F* (1, 35) = 175.91	<0.001	0.08
Trial Type	*F* (2, 70) = 50.75	<0.001	0.59
SOA	*F* (3, 105) = 5.70	<0.001	0.14
Modality × SOA	*F* (3, 105) = 6.57	<0.001	0.16
Modality × Trial Type	*F* (2, 70) = 2.94	=0.059	0.08
SOA × Trial Type	*F* (6, 210) = 5.50	<0.001	0.14
Modality × SOA × Trial Type	*F* (6, 210) = 13.01	<0.001	0.27
Attend to Visual			
Trial Type	*F* (2, 70) = 27.56	<0.001	0.44
SOA	*F* (3, 105) = 15.17	<0.001	0.30
SOA × Trial Type	*F* (6, 210) = 6.96	<0.001	0.17
Attend to Auditory			
Trial Type	*F* (2, 70) = 24.36	<0.001	0.41
SOA	*F* (3, 105) = 0.37	=0.772	0.01
SOA × Trial Type	*F* (6, 210) = 11.33	<0.001	0.24

## Data Availability

The datasets analyzed during the current study are available upon reasonable request.

## Appendix A

List of auditory and visual stimuli included in Experiments 1 and 2.

**Stimuli**	**Visual**	**Auditory**	**Study Phase**	**Category**	**Experiment**
Elephant	x	x	Practice	Animal	Exp 1 & 2
Motorcycle	x	x	Practice	Vehicle	Exp 1 & 2
Dog	x	x	Test	Animal	Exp 1 & 2
Cat	x	x	Test	Animal	Exp 1 & 2
Frog	x	x	Test	Animal	Exp 1 & 2
Monkey	x	x	Test	Animal	Exp 1 & 2
Car	x	x	Test	Vehicle	Exp 1 & 2
Boat	x	x	Test	Vehicle	Exp 1 & 2
Train	x	x	Test	Vehicle	Exp 1 & 2
Bike	x	x	Test	Vehicle	Exp 1 & 2
Violin		x	Test	Irrelevant	Exp 1
Cymbal		x	Test	Irrelevant	Exp 1
Bowling strike		x	Test	Irrelevant	Exp 1
Cash register		x	Test	Irrelevant	Exp 1
Bubbles		x	Test	Irrelevant	Exp 1
Hammer		x	Test	Irrelevant	Exp 1
Pinball		x	Test	Irrelevant	Exp 1
Sawing		x	Test	Irrelevant	Exp 1
Banana	x		Test	Irrelevant	Exp 1
Balloon	x		Test	Irrelevant	Exp 1
Comb	x		Test	Irrelevant	Exp 1
Clock	x		Test	Irrelevant	Exp 1
Broom	x		Test	Irrelevant	Exp 1
Watermelon	x		Test	Irrelevant	Exp 1
Scissors	x		Test	Irrelevant	Exp 1
Apple	x		Test	Irrelevant	Exp 1
Green circle	x		Test	Attention Check	Exp 1 & 2
Phone ring		x	Test	Attention Check	Exp 2
